# Hospitals and General Practitioners

**Published:** 1904-02-20

**Authors:** 


### Hospitals and General Practitioners.

It must, we fear, be admitted that hospitals do not invariably hold a secure position in the affections of those engaged in general practice in their neighbourhood. Occasions for annoyance and irritation readily arise and even a few of these are sufficient to breed a general attitude of suspicion and mistrust. There are according to existing arrangements no ready opportunities for removing misunderstandings, and often, therefore, these remain, and with them hostility and bitterness. That such results are much to be deplored will be admitted by all. Unfortunate in themselves they carry the further regret that they substitute themselves for what ought to be a relationship of mutual helpfulness and goodwill. When the attempt is made to fix responsibility for this state of matters the task is not found to be an easy one. Doubtless there are faults on both sides. But inquiry may show that with comparatively little trouble a relationship may be established that will work to the benefit of all concerned. It may be suggested without offence that not infrequently the practitioner has a legitimate grievance against some members of the hospital staff. His diagnosis and treatment in individual cases come under review in his absence, and there is not always observed that generous tone of criticism which both fair play and courtesy demand. This remark no doubt applies more especially to the younger members of the hospital organisation but even so it becomes the seniors to illustrate and enforce a high standard of action in this respect, and to remind their subordinates of the necessity for a considerate reticence. The claims of professional etiquette are as strong in the case of hospital patients as they are in private practice, and it is to everyone's advantage that they should be recognised and enforced. Again, it may be suggested that the position of the practitioner should receive full justice when a patient who has previously been under his treatment comes within the care of the hospital staff. He certainly ought to be apprised of the time when the case will be investigated and he should be invited to attend to contribute his share to the formation of a diagnosis and to the preparation of a scheme of treatment. Of course the urgency of the position or other circumstances may make all this at times impossible even in the in-patient department and in out-patient practice it can only be adopted in occasional instances. But even though it be condemned as a counsel of perfection it is an ideal which may usefully be kept in mind, and, if honestly pursued, would go far to remove from the mind of the practitioner the sense of injustice under which he not infrequently labours. A courteous note which at the worst can be dictated in a few minutes would be a practical recognition of the practitioner's position and would be appreciated by him as in harmony with the best traditions of professional life. It must not be imagined that the benefits of such a proceeding would be purely one-sided. Many difficulties and obscurities arising in hospital practice may be resolved by consultation with the practitioner who has seen the earlier phases of the case, and this is obviously to the advantage of the patient, whose interest after all is the chief one to be considered. There is no need to enlarge on the general improvement in mutual respect and regard which such a policy would inevitably produce. It would give repeated opportunities for the removal of misunderstandings and for the recognition of difficulties. It would also help the hospital to grow into a centre of worthy professional and clinical influence, and would make it recognised by medical men in the neighbourhood as affording an opportunity for real helpfulness in cases of difficulty and for personal education. There probably never was a time in the history of the profession when the desire for self-efficiency was so keen as it is at present. The smaller hospitals being free from the responsibility of teaching students have a great chance to contribute to the satisfaction of this desire. The staffs of such hospitals will be wise to recognise this. By cultivating fair and proper relationships with neighbouring practitioners they may not only render justice to their professional brethren but may utilise their positions for the advancement of post-graduation education and efficiency. The good sense and reasonableness, however, must not be on one side only. Hospital staffs suffer under difficulties and unjust criticisms not less than private practitioners. What the circumstances require is a willingness to recognise the true facts of the situation and to apply to events a generous and charitable construction. And these like many other desirable ends are most likely to be secured by the observance of the usual professional courtesies and by that mutual discussion in which men generally discover that even their opponents are not so black as rumour has painted them.

